# A perfect palindrome in the *Escherichia coli* chromosome forms DNA hairpins on both leading- and lagging-strands

**DOI:** 10.1093/nar/gku1136

**Published:** 2014-11-11

**Authors:** Benura Azeroglu, Frédéric Lincker, Martin A. White, Devanshi Jain, David R.F. Leach

**Affiliations:** Institute of Cell Biology, School of Biological Sciences, University of Edinburgh, King's Buildings, Mayfield Road, Edinburgh, EH9 3BF, UK

## Abstract

DNA palindromes are hotspots for DNA double strand breaks, inverted duplications and intra-chromosomal translocations in a wide spectrum of organisms from bacteria to humans. These reactions are mediated by DNA secondary structures such as hairpins and cruciforms. In order to further investigate the pathways of formation and cleavage of these structures, we have compared the processing of a 460 base pair (bp) perfect palindrome in the *Escherichia coli* chromosome with the same construct interrupted by a 20 bp spacer to form a 480 bp interrupted palindrome. We show here that the perfect palindrome can form hairpin DNA structures on the templates of the leading- and lagging-strands in a replication-dependent reaction. In the presence of the hairpin endonuclease SbcCD, both copies of the replicated chromosome containing the perfect palindrome are cleaved, resulting in the formation of an unrepairable DNA double-strand break and cell death. This contrasts with the interrupted palindrome, which forms a hairpin on the lagging-strand template that is processed to form breaks, which can be repaired by homologous recombination.

## INTRODUCTION

Palindromic DNA sequences contain the same order of bases on both strands, taking into account the antiparallel polarity of the molecule. This means that a single-strand of a DNA palindrome is self-complementary and can form a hairpin structure. If two such hairpins form opposite to each other, a cruciform structure can be generated. These hairpins and cruciforms are understood to mediate the biological consequences of DNA palindromes. In mammalian cells, there is evidence for extrusion and processing of cruciform structures that are responsible for constitutive translocations occurring at palindromic AT-rich repeats (PATRR) (1–3). In yeast cells, a pathway has been proposed where cruciform extrusion is followed by nuclease cleavage that leads to dicentric and acentric inverted duplications (4–6). In *Escherichia coli* (7) and in mammalian cells (8), this rearrangement pathway is not mediated by cruciform structures but by DNA hairpins. Furthermore, in *E. coli*, there is evidence for a pathway of hairpin cleavage following DNA replication by the SbcCD nuclease, which results in a DNA double-strand break (DSB) that can be repaired by recombination with an unbroken sister chromosome (9).

So far, there is no strong evidence for a cruciform processing pathway in bacteria, despite palindromic DNA sequences being less well tolerated than they are in eukaryotic cells, where evidence for cruciform extrusion is stronger. This paradox seems all the more acute given that bacteria have negatively supercoiled genomes and that negative supercoiling is required to favour cruciform extrusion *in vitro* (10). There might be two reasons explaining this paradox. First, if cruciform extrusion results in cell death, the reaction may be hard to observe. Second, the reaction may not occur because of the substantial kinetic barrier to cruciform extrusion, which is well understood from *in vitro* studies (11–13). Some indirect evidence consistent with the first explanation has been obtained for perfect and near-perfect palindromes in bacteriophage lambda during lytic growth in *E. coli* (14,15), while some evidence in favour of the second hypothesis comes from the observation that only palindromes with AT-rich central sequences, predicted to have low kinetic barriers to extrusion, have been detected as cruciforms *in vivo* in *E. coli* (16–21). However, none of these studies describe a clear pathway for the processing of cruciform structures that would be analogous to the SbcCD-mediated cleavage of hairpin structures formed during replication. Cruciform extrusion *in vitro* of sequences (other than highly AT-rich DNA) is initiated via the formation of a proto-cruciform by melting of the intra-strand base pairs of the central sequence of a DNA palindrome in a salt-dependent reaction, followed by branch migration to fully extrude the structure (11–13). We were therefore interested to know whether a similar reaction might occur *in vivo* leading to cell death caused by DNA cleavage and so set out to investigate the pathway of processing of a perfect palindrome in the context of the *E. coli* chromosome.

Previous work has established that *E. coli* cells, containing in their chromosomal *lacZ* gene a 246 bp interrupted palindrome with a 24 bp asymmetric central sequence separating inverted repeats of 111 bp, behave *in vivo* as if the palindrome forms a hairpin on the single-stranded lagging-strand template (9). This is inferred by the observation that while recombination proficient cells tolerate this palindrome without any noticeable problem even in the presence of SbcCD, recombination deficient cells die as a consequence of SbcCD expression. We concluded that a hairpin is formed and is cleaved on one and only one of the two sister chromosomes, allowing efficient repair by recombination in wild-type cells but causing death in the absence of recombination. Indeed, when SbcCD is expressed in *recB* mutant cells containing the 246 bp palindrome, a two-ended DNA DSB is detected in at least 35% of chromosomes, close to the 50% predicted if every lagging-strand were cleaved by SbcCD (9). It is clear that a central spacer of 24 bp would provide a substantial kinetic barrier to the extrusion of a cruciform structure (12,13) and this is consistent with the detection of a hairpin only in single-stranded DNA.

Since our previous work (14,15) implied that a perfect palindrome might have the potential to form a cruciform structure *in vivo*, we reasoned that this structure might be cleaved at its two hairpin loops by SbcCD and/or at its 4-way junction by a Holiday junction endonuclease, such as RuvABC. The viability of strains containing this perfect palindrome and lacking SbcCD, RuvAB or both would reveal the protein(s) responsible of the cleavage of this potential cruciform structure. Cleavage of a cruciform structure would result in cell death irrespective of the recombination proficiency of the strain as both DNA strands would be cleaved, leaving no template for repair by recombination. Using a conditional expression system for SbcCD, we were able to construct a strain carrying a perfect palindrome of 460 bp in the absence of this hairpin endonuclease. However, in the presence of SbcCD, even recombination proficient cells died. Death in a *rec***^+^** host following cleavage by SbcCD was suggestive of cruciform extrusion but the lack of any effect of the Holliday junction endonuclease RuvABC made us wonder whether this interpretation was correct. We have therefore carried out further studies to understand the cleavage of this DNA sequence and we present evidence that DNA replication causes the formation of two hairpin DNA structures opposite each other and not of a cruciform structure. This work reveals that a perfect palindrome can form a hairpin DNA structure on the leading-strand template as well as the lagging-strand template of the replication fork.

## MATERIALS AND METHODS

### Oligonucleotides, bacteriophages, plasmids and strains used

All oligonucleotides, bacteriophages, plasmids and strains used are listed in the Supplementary Data, Supplementary Tables S1–S4. Chromosomal alterations were introduced by P1 transduction or by plasmid-mediated gene replacement (PMGR) (22,23). Plasmids for PMGR were pTOF24 derivatives (23). Construction details, including sequences of oligonucleotides used, are provided in the Supplementary Data.

### Cell viability assays

To measure the viability of strains by spot tests, 10-fold serial dilutions of overnight cultures were prepared and 3 μl of these dilutions were spotted onto LB agar plates supplemented with 0.2% arabinose or 0.5% glucose. These experiments were carried out three independent times, giving similar results.

To measure growth rates, cells were grown at 37ºC under agitation in LB medium supplemented with the indicated sugar. Overnight cultures of individual strains were diluted and maintained in exponential growth phase by appropriate dilutions at regular intervals. Optical density (OD_600nm_) was maintained below 0.5 and growth was monitored by measuring the OD_600nm_.

### Induction of DSBs and isolation of chromosomal DNA in agarose plugs

Overnight cultures grown in 5 ml LB medium were diluted to an OD_600nm_ of 0.02 and grown at 37°C with agitation up to an OD_600nm_ of 0.2. Expression or repression from the P*_BAD_-sbcDC* construct was obtained by adding 0.2% arabinose or 0.5% glucose, respectively. If necessary, the culture was split in two and 0.2% arabinose or 0.5% glucose were added to the respective half of the initial culture. Cultures were put back at 37°C to grow for 1 h. Notably, the experiments using the *dnaA*^ts^ mutants, to demonstrate replication dependence of the cleavage were carried out as follows. A culture grown at 30°C overnight was diluted and grown at 30°C under agitation up to an OD_600nm_ of 0.2 when it was then shifted to 42°C for 2 h. Then, the culture was split in two and 0.5% glucose or 0.2% arabinose were added. Cultures were put back at 42°C to grow for 1 h. Cells were harvested at 4°C and washed three times in TEN buffer (50 mM Tris, 50 mM ethylenediaminetetraacetic acid (EDTA), 100 mM NaCl, pH 8.0). Cells were re-suspended in TEN buffer to an OD_600nm_ of six and mixed with an equal volume of 2% low melting point agarose (Invitrogen) prepared in TEN buffer and equilibrated to 37°C. The mix was poured into plug moulds (BioRad) and allowed to set for an hour. Plugs were treated in NDS solution (0.5 M EDTA, 10 mM Tris, 0.55 M NaOH, 36.8 mM lauroyl sarcosine; pH 8.0) supplemented with 1 mg/ml of proteinase K (Roche) and put at 37°C overnight under agitation. Fresh NDS + proteinase K were added for a second overnight after which plugs were stored at 4°C in fresh NDS. Before digestion of the DNA, a plug was washed in 1X restriction buffer six times, replacing the buffer every hour. The plug was then placed in fresh 1X restriction buffer, supplemented with the restriction enzyme and incubated at 37°C overnight, rocking.

### Agarose gel electrophoresis

A plug in which the DNA was digested with a restriction enzyme was run in 0.5X TBE (89 mM Tris-borate, 2 mM EDTA) on a 1% (w/v) agarose gel at 2 V/cm for 12 h at 4°C. The DNA was transferred to a positively charged nylon membrane by Southern blotting and cross-linked using ultraviolet-light.

### Radioactive detection of DNA

DNA was detected using ^32^P α-dATP incorporated (using Stratagene Prime-It II random primer labelling kit) into a polymerase chain reaction (PCR) fragment. Probes were hybridized to membranes overnight at 65°C in 10 ml of Church-Gilbert buffer (7% sodium dodecyl sulphate (SDS), 0.5 M NaH_2_PO_4_, 1 mM EDTA, 1% bovine serum albumin). Membranes were washed at 60°C in 2X SSC (1X SSC: 0.15 M NaCl, 0.015 M Na-citrate), supplemented with 0.1% SDS, for 15 min and then 0.5X SSC, supplemented with 0.1% SDS, for 30 min. Labelled membranes were exposed to GE healthcare storage phosphor screens and scanned using a Molecular Dynamics Typhoon FLA 7000 phosphor imager scanner. Images were quantified using GE healthcare ImageQuant TL.

### Gel analysis

For the purpose of quantification, the data obtained from *lacZ* probing were normalized to the data obtained from *cysN* probing. Then, the data were normalized to either T0 (for *rec**^+^*** and *recB* gels) or no palindrome (for replication dependence assay) control. Background signal was subtracted prior to normalizations.

## RESULTS

### SbcCD-mediated cleavage of a 460 bp perfect palindrome causes cell death in a recombination proficient host

**Figure 1.**
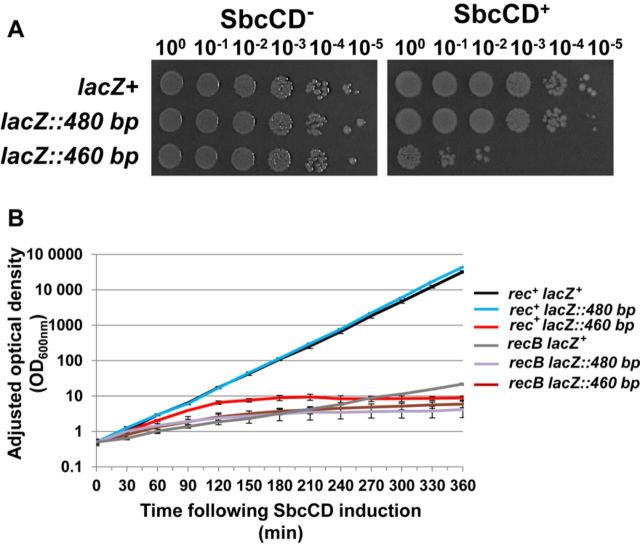
Perfect palindrome induced breaks cause death in recombination-proficient cells. (**A**) Colony formation of 10-fold dilutions of a strain containing the perfect 460 bp palindrome was compared to 10-fold dilutions of a strain without a chromosomal palindrome and of a strain containing a 480 bp interrupted palindrome. In the left panel, cells were incubated in the presence of glucose to repress the expression of SbcCD and in the right panel, cells were incubated in the presence of arabinose to induce the expression of SbcCD. (**B**) The optical density of the same three strains and their *recB* mutant derivatives was monitored as a function of time after addition of arabinose to induce the expression of SbcCD. Cells were maintained in exponential phase by dilution and the plotted optical density was adjusted for that dilution. The strains used were DL2792 (*lacZ^+^*), DL3020 (*lacZ::460 bp*), DL3021 (*lacZ::480 bp*), DL2797 (Δ*recB lacZ^+^*) DL3066 (Δ*recB lacZ::460 bp*) and DL3048 (Δ*recB lacZ::480 bp*). Error bars represent the standard error of the mean of three biological replicates.

A perfect palindrome of 460 bp (230 bp per arm) was inserted in the *lacZ* locus of the *E. coli* chromosome in a strain with the *sbcDC* genes under the control of the arabinose inducible P_*araBAD*_ promoter. The growth of these cells was normal in a medium containing glucose, which represses the expression of SbcCD (Figure 1). However, when expression of SbcCD was induced using arabinose, growth was arrested and cells died despite the recombination proficiency of the host. This behaviour is radically different to that of the previously studied 246 bp interrupted palindrome (which has a 24 bp central asymmetry), where a similar chromosomal insertion caused loss of viability only in recombination deficient hosts (9). To test whether cell death in a recombination proficient host was caused by the length or the perfection of the new palindrome, we compared the behaviour of strains containing the 460 bp perfect palindrome with strains containing a 480 bp interrupted palindrome consisting of the same 230 bp arms separated by a central asymmetry of 20 bp. Here, as for the 246 bp interrupted palindrome, death was only observed following SbcCD expression in recombination defective hosts (Figure 1). In the presence of attempted repair (*rec*^+^) there is strong induction of the SOS response associated with severe filamentation of the cells with the perfect 460 bp palindrome. This filamentation is associated with the initial increase in OD, which plateaus at ∼120 min. In recombination proficient cells with the interrupted 480 bp palindrome, there is modest SOS induction and modest filamentation but the filaments divide without significant loss in viability and no plateau in the OD measurement (see videos 1–3 in Supplementary Materials).

**Figure 2.**
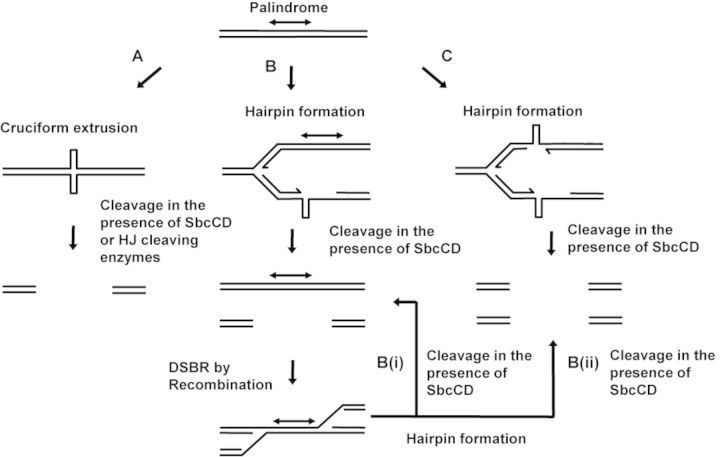
Hypotheses to explain the death caused by SbcCD cleavage of a perfect palindrome. (**A**) It has been suggested that there is a pathway of cruciform extrusion *in vivo*. However, the evidence is either indirect or involves very AT rich sequences. How these structures are processed, if they are formed, is unknown but they would be expected to be sensitive to cleavage by SbcCD and enzymes recognizing Holliday junctions (HJs). (**B**) SbcCD cleavage of a hairpin DNA structure formed during DNA replication leading to a two-ended DSB is repaired by recombination with a sister chromosome. Cleavage at an interrupted palindrome results in efficient repair. However, cleavage at a perfect palindrome might interfere with productive repair by either of the pathways B(i) or B(ii). In pathway B(i), the hairpin reforms during repair on only one of the sister chromosomes and death is caused by an endless cycle of cleavage, repair and re-cleavage. In pathway B(ii), hairpins form on both the sister chromosomes during repair and cleavage results in loss of any repair template. (**C**) Hairpins are formed on both the leading and lagging-strand templates during chromosomal DNA replication. Cleavage of both hairpins by SbcCD results in no template for repair.

We concluded that central symmetry is responsible for death in a recombination proficient host. This observation would be consistent with several hypotheses (Figure 2). (i) A perfect palindrome could be extruded into a cruciform structure that would be cleaved by SbcCD. (ii) As for an interrupted palindrome, a perfect palindrome could only form a hairpin on one of the two templates, but the repair of the DSB would lead to re-cleavage of a newly-formed hairpin structure. Two versions of this second hypothesis would be possible. In the first, re-cleavage during repair would occur on only one of the two sister chromosomes, inducing a never-ending cycle of cleavage and repair that eventually would cause cell death, and in the second re-cleavage would occur on both chromosomes causing death by the loss of templates for repair. (iii) A perfect palindrome could form hairpin DNA structures that would be cleaved by SbcCD on both the leading- and lagging-strand templates.

### Replication-dependent DSBs, induced by SbcCD at a perfect palindrome are detected in a recombination proficient host

**Figure 3.**
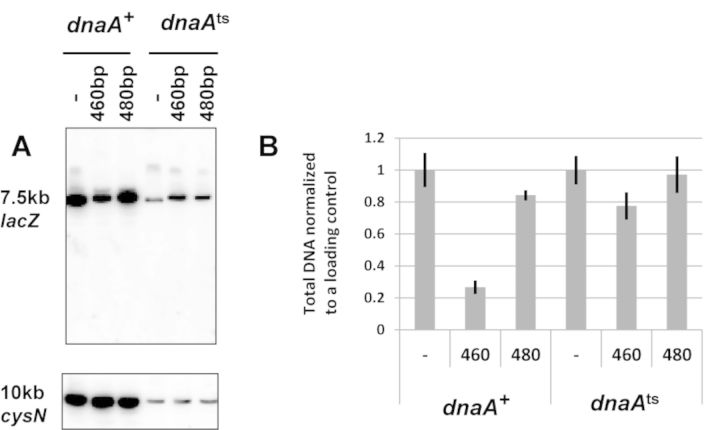
Replication-dependence of DNA loss surrounding the break in recombination-proficient palindrome-containing cells. (**A**) Top panel. Southern blot showing the 7.5 kb NdeI *lacZ* fragment containing the palindrome. Lower panel. Southern blot showing the 10 kb NdeI *cysN* fragment (it can be seen that incubation at 42°C reduces the total yield of DNA). (**B**) Quantification of DNA loss. The total amount of DNA from each sample was quantified. DNA samples were taken after 60 min of growth in arabinose to induce expression of SbcCD. Strains used were DL2792 (*lacZ^+^*), DL3020 (*lacZ*::*460bp*) and DL3021 (*lacZ*::*480bp*), DL3584 (*dnaA*^ts^*lacZ^+^*), DL3585 (*dnaA*^ts^*lacZ*::*460bp*) and DL3586 (*dnaA*^ts^*lacZ*::*480bp*). Error bars represent the standard error of the mean of three biological replicates. The difference in yields of DNA recovered between the *dnaA*^ts^ strains containing the 460-bp perfect palindrome and the 480 bp interrupted palindrome at 42°C was not statistically significant (unpaired two-tailed *t*-test *p* = 0.074).

Death of recombination-proficient cells containing a 460 bp perfect palindrome was consistent with cleavage of all the DNA strands at the *lacZ* locus removing any template for DSB repair (DSBR). We tested this prediction by Southern hybridization following cleavage of the chromosome with NdeI and agarose gel electrophoresis (Figure 3). A fragment of 7.4 kb containing the palindrome was probed with a PCR fragment complementary to the *lacZ* gene and a fragment of 10 kb from the other side of the chromosome was probed with a PCR fragment complementary to *cysN*, in order to normalize for gel loading. The DNA fragment containing the palindrome is clearly lost even though the cells are recombination proficient. This was consistent with cruciform extrusion of this DNA sequence. However, this explanation was weakened by the observation that RuvABC, the Holliday junction resolvase of *E. coli*, previously shown to cleave cruciform structures (24), was not responsible for cell death, as we were able to construct a viable strain harbouring the perfect palindrome in the presence of *ruvABC* genes. Cruciform extrusion *in vitro* occurs in negatively supercoiled DNA without any requirement for DNA replication. We therefore tested the replication-dependence of perfect palindrome cleavage. To do this, we tested DNA loss in a *dnaA*^ts^ mutant at 42°C where DNA replication is prevented. As seen in Figure 3, DNA loss in a strain containing the 460 bp palindrome is replication dependent. Therefore, SbcCD-mediated cleavage of the 460 bp palindrome is not due to a cruciform structure extruded from unreplicated supercoiled DNA. Coupled to the lack of cleavage by the Holliday junction resolving enzyme RuvABC, this result provides further evidence against a cruciform extrusion pathway.

### Predictions of hairpin cleavage models

**Figure 4.**
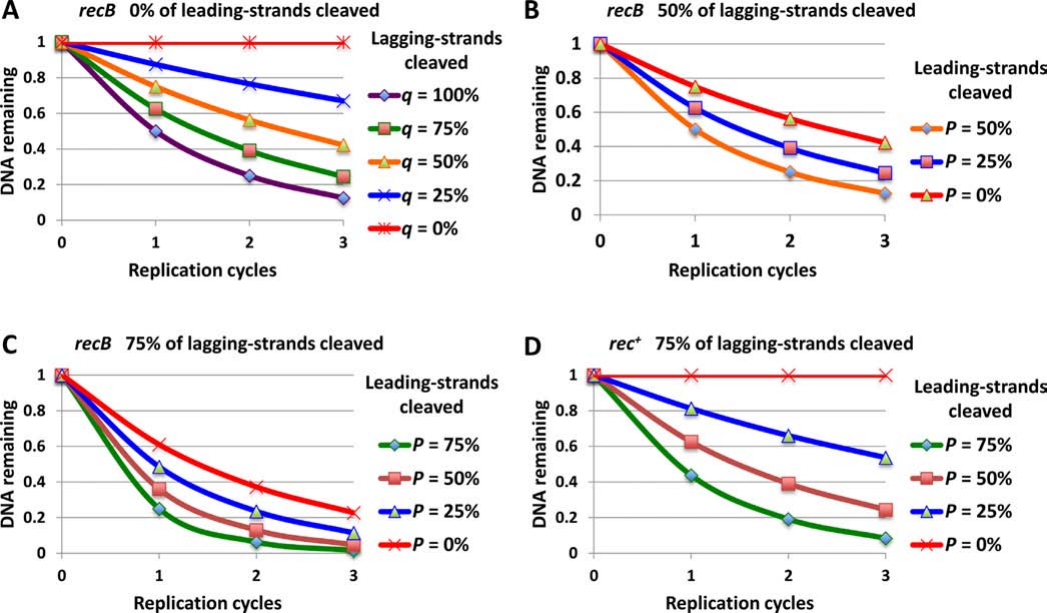
Predictions for DNA loss of alternative probabilities for cleaving leading- and lagging-strands during DNA replication. Expected DNA losses are presented for cleavage probabilities of 0, 25, 50, 75 and 100% as explained below. (**A**) Predicted DNA loss for cleavage of the lagging-strand in the absence of DSBR (*recB* mutant). This is the behaviour we observe for an interrupted palindrome (9) and the DNA loss we observe corresponds a frequency of strand-cleavage between 50 and 75%. We have therefore predicted (in **B** and **C**) the DNA loss obtained when both strands are cleaved using these two alternative probabilities of lagging-strand cleavage. (B and C) Predicted DNA loss for a 50% (B) and 75% (C) probabilities of lagging-strand cleavage coupled to probabilities of leading-strand cleavage of 0, 25, 50 and 75% in the absence of DSBR (*recB* mutant). In both cases we have assumed that the probability of leading-strand cleavage can be equal to that for lagging-strand cleavage but cannot exceed it. Our data are consistent with cleavage of both the leading- and lagging-strands of between 50 and 75%. (**D**) Predicted DNA loss for a 75% probability of lagging-strand cleavage coupled to probabilities of leading-strand cleavage of 0, 25, 50 and 75% in the presence of DSBR (rec^+^ cells). DNA is not expected to be lost in recombination proficient cells if the leading strand is not cleaved (0%). However, if it is cleaved, there will be no template for repair and DNA will be lost. Here, we have only illustrated the situation for 75% cleavage of the lagging-strand because the observed loss after three generations for the perfect palindrome corresponds to that predicted for cleavage of 75% of both strands.

The lack of evidence for a cruciform extrusion pathway that could explain SbcCD-mediated killing of recombination proficient cells containing a perfect palindrome led us to consider two alternative possibilities. Either death of a recombination proficient host was caused by cleavage of hairpins formed on both leading- and lagging-strand templates or the cause of cell death was re-cleavage of the products of DSBR. We reasoned that these hypotheses would result in differences in the proportions of chromosomes cleaved in *rec***^+^** and *recB* mutant cells. Therefore, we predicted the proportion of DNA strands remaining following chromosome cleavage in the presence or absence of DSBR. In a recombination proficient host, loss of the DNA fragment containing the palindrome is expected to occur only if both replicated copies of the chromosome are cleaved, whereas in a *recB* mutant host loss of all broken chromosomes is expected. We therefore estimated the proportion of DNA remaining (*R***^+^**) per replication cycle in a *rec***^+^** host to be *R***^+^** = 1 −*pq*, where *p* is the probability of cleaving the leading-strand and *q* is the probability of cleaving the lagging-strand. Similarly, we estimated the proportion of DNA remaining in a *recB* mutant host to be *R***^−^** = (*p* + *q*)/2. These estimations led to the predicted patterns of DNA loss shown in Figure 4.

### SbcCD-mediated cleavage of chromosomes containing a perfect palindrome does not require DSBR

**Figure 5.**
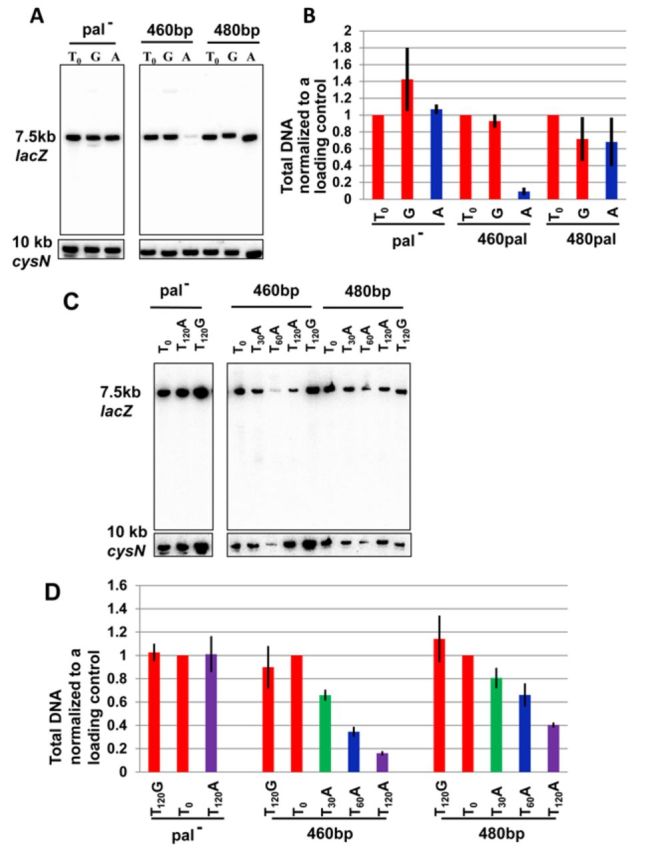
(**A** and **B**) Quantification of DNA loss surrounding the break in palindrome-containing and palindrome-free recombination proficient cells. (A) Top panel. Southern blot showing the 7.5 kb NdeI *lacZ* fragment containing the palindrome. Lower panel. Southern blot showing the 10 kb NdeI *cysN* fragment. (B) Quantification of DNA loss. The total amount of DNA from each sample was quantified and normalized to the total DNA for the 10 kb NdeI *cysN* fragment on the opposite side of the chromosome. Error bars represent the standard error of the mean of three biological replicates. Strains used were DL2792 (*lacZ^+^*), DL3020 (*lacZ*::*460bp*) and DL3021 (*lacZ*::*480bp*). The DNA samples were taken before (T0) and 60 min after the induction of SbcCD expression with arabinose (A) and 60 min after the repression of SbcCD expression with glucose (G). (**C** and **D**) Quantification of DNA loss surrounding the break in palindrome-containing and palindrome-free recombination deficient cells. (C) Top panel. Southern blot showing the 7.5 kb NdeI *lacZ* fragment containing the palindrome. Lower panel. Southern blot showing the 10 kb NdeI *cysN* fragment. (D) Quantification of DNA loss. The total amount of DNA from each sample was quantified and normalized to the total DNA for the 10 kb NdeI *cysN* fragment on the opposite side of the chromosome. Error bars represent the standard error of the mean of three biological replicates. Strains used were DL2797 (Δ*recB lacZ^+^*), DL3066 (Δ*recB lacZ*::*460bp*) and DL3048 (Δ*recB lacZ*::*480bp*). The DNA samples were taken before (T0) and 30, 60 and 120 min after the induction of SbcCD expression with arabinose (T30A, T60A and T120A) and 120 min after the repression of SbcCD expression with glucose (T120G).

As can be seen in Figure 5A and B, the vast majority of the fragment containing the perfect palindrome was lost in a *rec***^+^** host after 60 min growth in the presence of SbcCD. As the cells were grown with a generation time of 23 min this DNA loss occurred over approximately three generations. Quantification of the signals (following normalization for loading) indicated that 92% of the *lacZ* fragment containing the 460 bp perfect palindrome was lost. By contrast, no significant loss of the same fragment containing the 480 bp interrupted palindrome was detected. These data confirm that the perfect palindrome is cleaved on both templates for the leading- and lagging-strands but do not distinguish between cleavage during chromosomal DNA replication or during repair.

In order to distinguish whether the SbcCD mediated cleavage of a perfect palindrome on both strands is occurring during replication or repair, we measured DNA fragment loss at the palindrome in a *recB* mutant where DSBR is abrogated and the generation time was 56 min. As can be seen in Figure 5C and D, DNA loss was substantial, amounting to 35, 66 and 84 for the perfect palindrome, following 30, 60 and 120 min of SbcCD induction, respectively. By contrast, losses of 20, 34 and 60% were observed following 30, 60 and 120 min of SbcCD induction in the control strain containing the 480 bp interrupted palindrome. Clearly, the loss of DNA for the strain containing the perfect palindrome was greater than for the strains with either of the interrupted palindromes. This is consistent with cleavage of both leading-and lagging strands during DNA replication and not during DSBR.

## DISCUSSION

We have shown that both recombination proficient and recombination deficient *E. coli* cells containing a 460 bp perfect palindrome inserted in the chromosome die in the presence of the SbcCD hairpin nuclease. This behaviour is radically different to that of a control strain containing a 480-bp interrupted palindrome, which only dies when SbcCD is expressed in recombination deficient cells. DNA loss of the fragment containing the perfect palindrome is observed in a recombination proficient host and this loss is not observed for the interrupted palindrome. This is consistent with cleavage of all the DNA strands by SbcCD as expected if the perfect palindrome is able to extrude into a cruciform structure or DNA hairpins are formed on both the leading- and lagging-strand templates during DNA replication. This cleavage explains death in a recombination proficient host by removal of the template for DSBR.

Cleavage of the perfect palindrome is replication-dependent arguing against replication-independent cruciform extrusion. This led us to test whether cleavage was occurring during chromosomal DNA replication or during repair DNA synthesis subsequent to chromosomal replication. DNA loss is enhanced by palindrome symmetry in a *recB* host that is defective for DSBR, arguing that elevated cleavage of the perfect palindrome is not dependent on repair. Comparing the DNA losses observed for the interrupted palindromes in the *rec***^+^** and *recB* strains (Figure 5) with the predicted losses (Figure 4), we conclude that between 50 and 75% of lagging-strands are cleaved per generation. Similarly, the losses observed for the perfect palindrome are consistent with cleavage of between 50 and 75% of both the leading- and lagging-strands per generation.

Our results suggest that there is enough single-stranded DNA between the replicative helicase and the leading-strand DNA polymerase to initiate misfolding. Nevertheless, short perfect palindromes are common in the chromosome, so it must be that the long arms of the sequence come into play at some stage to generate a structure that is a substrate for cleavage by SbcCD. *In vitro* the lower size limit for hairpin cleavage by SbcCD corresponds to structures that would be formed by palindromes of between 20 and 36 bp in length, ensuring that naturally occurring genomic palindromes are not targeted (25). Elongation of the hairpin, following the formation of a small nucleating structure, may involve the displacement of the newly synthesized leading-strand. It is interesting to note that a G-quadruplex forming sequence in yeast causes more instability when the G-rich strand is the template for the leading-strand (26). This may reflect a similar accessibility of the DNA between the replicative helicase and leading-strand DNA polymerase in eukaryotic cells.

## SUPPLEMENTARY DATA

Supplementary Data are available at NAR Online.

SUPPLEMENTARY DATA
