# Supplementary material for: A perfect palindrome in the Escherichia coli chromosome forms DNA hairpins on both leading- and lagging-strands
Source: Nucleic Acids Res. 2014 Nov 11;42(21):13206–13. doi: 10.1093/nar/gku1136 (PMC4245961; doi:10.1093/nar/gku1136)
Supplement: SUPPLEMENTARY DATA [file supp_42_21_13206__index.html]

A perfect palindrome in the Escherichia coli chromosome forms DNA hairpins on both leading- and lagging-strands — SUPPLEMENTARY DATA 

# A perfect palindrome in the *Escherichia coli* chromosome forms DNA hairpins on both leading- and lagging-strands

## SUPPLEMENTARY DATA

**Files in this Data Supplement:**

- SUPPLEMENTARY DATA
- SUPPLEMENTARY DATA
