# Supplementary material for: A perfect palindrome in the Escherichia coli chromosome forms DNA hairpins on both leading- and lagging-strands
Source: Nucleic Acids Res. 2014 Nov 11;42(21):13206–13. doi: 10.1093/nar/gku1136 (PMC4245961; doi:10.1093/nar/gku1136)
Supplement: SUPPLEMENTARY DATA [file supp_gku1136_nar-02535-f-2014-File007.docx]

***Supplementary Data:***

**A Perfect Palindrome in the *Escherichia coli* Chromosome Forms DNA Hairpins on both Leading- and Lagging-Strands**

**Benura Azeroglu, Frédéric Lincker, Martin A. White, Devanshi Jain and David R. F. Leach**

Institute of Cell Biology, School of Biological Sciences, University of Edinburgh, King’s Buildings, Mayfield Road, Edinburgh, EH9 3JR, UK

[D.Leach@ed.ac.uk](mailto:D.Leach@ed.ac.uk)

**Supplementary Methods**

**Plasmid Construction**

Plasmid pDL2010 was created in order to insert a 460 bp perfect palindrome into the *lacZ* locus of the *E. coli* chromosome. The palindrome was amplified from lambda DRL133 DNA using CF2 and CR1 primers and digested using EcoRI and inserted at an MfeI site in the pLacD1 plasmid ([1](#_ENREF_1)).

Plasmid pDL2179 was created in order to insert a 480 bp perfect palindrome into the *lacZ* locus of the *E. coli* chromosome. The palindrome was amplified from lambda DRL167 DNA using CF2 and CR1 primers and digested using EcoRI and inserted at an MfeI site in the pLacD1 plasmid ([1](#_ENREF_1)). The interrupted palindrome has the same arms as the perfect palindrome but contains a 20 mer interruption (5’- ACTTAGTGGATCTCAATTCA-3’).

Both plasmids were propagated in the *sbcC* mutant strain DL733, which is permissive for the propagation of palindromic DNA sequences.

**Visualisation of Filamentation by Time-Lapse Microscopy (2)**

Overnight cultures were diluted and these cultures grown at 37°C in LB medium to an OD600nm between 0.1 and 0.3. Then, 5 μl of culture was placed on an airtight LB-coated slide. Brightfield images were acquired every 30 seconds for 2 hours at a resolution of 129 nm per pixel using a Zeiss Axiovert 200 fluorescence microscope equipped with a Photometrics cool-SNAP HQ CCD camera. Images were analysed and films were constructed using the program MetaMorph 6-3r2 (Molecular Devices). The behaviour of three *rec^+^* strains was compared: DL2573 (no palindrome), DL2949 (480 bp interrupted palindrome) and DL2999 (460 bp perfect palindrome).

**Table S1: DNA oligonucleotide sequences used**

| **Name** | **Sequence (5' to 3')** | **Purpose** |
| --- | --- | --- |
| CF2 | ATACCCAGATTGCGAACACC | To amplify the palindromes from bacteriophage lambda |
| CR1 | ACAACCTGACCCAGCAAAAG |  |
| *lacZ*p.F | TAGCGGCTGATGTTGAACTG | To make the *lacZ* probe |
| *lacZ*p.R | ATGAACGGTCTGGTCTTTGC |  |
| *cysN*.F | GTCGTTATGCAGCGATGAGA | To make the *cysN* probe |
| *cysN*.R | GCGGAAAGCATCCACATTAT |  |

**Table S2: Bacteriophages used**

| **Phage** | **Genotype** | **Source** |
| --- | --- | --- |
| DRL133 | λ pal460 *spi6* *cI857* χ° | ([3](#_ENREF_2)) |
| DRL216 | λ pal480 Δ*spi6* *cI857* χ+ | (4) |

**Table S3: Plasmids used**

| **Name** | **Brief Description** | **Source** |
| --- | --- | --- |
| pTOF24 | Cm^R^ Km^R^ Ts Suc^S^ | (5) |
| pLacD1 | pTOF + *lacZ* fragment with MfeI cloning site, Cm^R^ Km^R^ Ts Suc^S^ | ([1](#_ENREF_1)) |
| pDL2010 | pLacD1 + *pal460* | This work |
| pDL2179 | pLacD1 + *pal480* | This work |

**Table S4: Bacterial strains used**

| **Strain** | **Genotype** | **Source** |
| --- | --- | --- |
| BW27784 | Δ(*araD-araB*)*567* Δ(*araH-araF*)*570*(::*FRT*) Δ*araEp-532*::*FRT*  *φP_cp18_araE533* Δ(*rhaD-rhaB*)*568 hsdR514* Δ*lacZ478*(::*rrnB-3*) | (6) |
| STL8297 | MG1655 *F^-^ dnaA46^ts^ tna::*Tn*10 rph-1* | (7) |
| DL733 | *rpsL* *ara* Δ(*lac-proAB*) *φ*8*0dlacZ*Δ*M15* Δ*sbcCD*::Km^R^ | (8) |
| DL2792 | BW27784  *proA*::*ISceI_cs_ tsx*::*ISceI_cs_ P_BAD_-sbcDC lacZ^+^*  *cynX*::Gm^R^ *lacIq lacZχ****^-^**** | (9) |
| DL2797 | BW27784 *recB268*::Tn*10*  *proA*::*ISceI_cs_ tsx*::*ISceI_cs_*  *P_BAD_-sbcDC lacZ^+^  cynX*::Gm^R^ *lacIq lacZχ****^-^*** | (9) |
| DL3020 | BW27784 *proA*::*I-SceI_cs_ tsx*::*I-SceI_cs_ lacZ*::*pal460 P_BAD_-sbcDC*  *lacIq lacZχ****^-^*** | This work |
| DL3021 | BW27784 *tsx*::*I-SceI_cs_ proA*::*I-SceI_cs_ lacZ*::*pal480 P_BAD_-sbcDC*  *lacIq lacZχ****^-^*** | This work |
| DL3048 | BW27784 Δ*recB tsx*::*I-SceI_cs_ proA*::*I-SceI_cs_ lacZ*::*pal480*  *P_BAD_-sbcDC lacIq lacZχ****^-^*** | This work |
| DL3066 | BW27784 Δ*recB tsx*::*I-SceI_cs_ proA*::*I-SceI_cs_ lacZ*::*pal460*  *P_BAD_-sbcDC lacIq lacZχ****^-^*** | This work |
| DL3584 | BW27784 *dnaA46^ts^ tna*::Tn*10 proA*::*ISceI_cs_ tsx*::*ISceI_cs_*  *P_BAD_-sbcDC lacZ^+^  cynX*::Gm^R^ *lacIq lacZχ****^-^*** | This work |
| DL3585 | BW27784 *dnaA46^ts^ tna*::Tn*10 proA*::*ISceI_cs_ tsx*::*ISceI_cs_*  *P_BAD_-sbcDC lacZ*::*pal460 cynX*::Gm^R^ *lacIq lacZχ****^-^*** | This work |
| DL3586 | BW27784 *dnaA46^ts^ tna*::Tn*10 proA*::*ISceI_cs_ tsx*::*ISceI_cs_*  *P_BAD_-sbcDC lacZ*::*pal480 cynX*::Gm^R^ *lacIq lacZχ****^-^*** | This work |

* *lacZχ****^-^*** indicates that the Chi site in *lacZ* has been removed without affecting the amino acid sequence encoded by the gene.

**Supplementary Videos**

Video 1: DL2573 (no palindrome)

Video 2: DL2949 (480 bp interrupted palindrome)

Video 3: DL2999 (460 bp perfect palindrome)

**References**

1. Zahra, R., Blackwood, J.K., Sales, J. and Leach, D.R. (2007) Proofreading and secondary structure processing determine the orientation dependence of CAG x CTG trinucleotide repeat instability in Escherichia coli. *Genetics*, **176**, 27-41.

2. Darmon, E., Eykelenboom, J.K., Lopez-Vernaza, M., White, M.A. and Leach, D.R.F. (2014) Repair on the go: *E. coli* maintains a high proliferation rate while repairing a chronic DNA double-strand break. *PLoS ONE, in press*.

3. Chalker, A.F., Okely, E.A., Davison, A. and Leach, D.R.F. (1993) The Effects of Central Asymmetry on the Propagation of Palindromic DNA in Bacteriophage-Lambda Are Consistent with Cruciform Extrusion Invivo. *Genetics*, **133**, 143-148.

4. Allers, T. and Leach, D.R. (1995) DNA palindromes adopt a methylation-resistant conformation that is consistent with DNA cruciform or hairpin formation in vivo. *J Mol Biol*, **252**, 70-85.

5. Merlin, C., McAteer, S. and Masters, M. (2002) Tools for characterization of Escherichia coli genes of unknown function. *J Bacteriol*, **184**, 4573-4581.

6. Khlebnikov, A., Datsenko, K.A., Skaug, T., Wanner, B.L. and Keasling, J.D. (2001) Homogeneous expression of the P(BAD) promoter in Escherichia coli by constitutive expression of the low-affinity high-capacity AraE transporter. *Microbiology*, **147**, 3241-3247.

7. Sutera, V.A., Jr. and Lovett, S.T. (2006) The role of replication initiation control in promoting survival of replication fork damage. *Mol Microbiol*, **60**, 229-239.

8. Connelly, J.C. and Leach, D.R. (1996) The sbcC and sbcD genes of Escherichia coli encode a nuclease involved in palindrome inviability and genetic recombination. *Genes Cells*, **1**, 285-291.

9. Eykelenboom, J.K., Blackwood, J.K., Okely, E. and Leach, D.R. (2008) SbcCD causes a double-strand break at a DNA palindrome in the Escherichia coli chromosome. *Mol Cell*, **29**, 644-651.
